# Real-time measurement of intraocular pressure variation during automatic intravitreal injections: An ex-vivo experimental study using porcine eyes

**DOI:** 10.1371/journal.pone.0256344

**Published:** 2021-08-26

**Authors:** Ikjong Park, Han Sang Park, Hong Kyun Kim, Wan Kyun Chung, Keehoon Kim

**Affiliations:** 1 Department of Mechanical Engineering, POSTECH, Pohang, Korea; 2 Department of Ophthalmology, Kyungpook National University Hospital, Daegu, Korea; University of Missouri-Columbia, UNITED STATES

## Abstract

**Purpose:**

To measure needle insertion force and change in intraocular pressure (IOP) in real-time during intravitreal injection (IVI). The effects of needle size, insertion speed, and injection rate to IOP change were investigated.

**Methods:**

Needle insertion and fluid injection were performed on 90 porcine eyeballs using an automatic IVI device. The IVI conditions were divided according to needle sizes of 27-gauge (G), 30G, and 33G; insertion speeds of 1, 2, and 5 mm/s; and injection rates of 0.01, 0.02, and 0.05 mL/s. Insertion force and IOP were measured in real-time using a force sensor and a pressure transducer.

**Results:**

The peak IOP was observed when the needle penetrated the sclera; the average IOP elevation was 96.3, 67.1, and 59.4 mmHg for 27G, 30G, and 33G needles, respectively. An increase in insertion speed caused IOP elevation at the moment of penetration, but this effect was reduced as needle size decreased: 109.8–85.9 mmHg in 27G for 5–1 mm/s (p = 0.0149) and 61.8–60.7 mmHg in 33G for 5–1 mm/s (p = 0.8979). Injection speed was also related to IOP elevation during the stage of drug injection: 16.65 and 11.78 mmHg for injection rates of 0.05 and 0.01 mL/s (p < 0.001).

**Conclusion:**

The presented data offers an understanding of IOP changes during each step of IVI. Slow needle insertion can reduce IOP elevation when using a 27G needle. Further, the injection rate must be kept low to avoid IOP elevations during the injection stage.

## Introduction

Intravitreal injections (IVIs) have been widely used for the direct delivery of anti-vascular endothelial growth factor (VEGF) or steroids into the eye. By bypassing the blood-ocular barrier, IVI ensures an adequate drug concentration in the retina. The IVI procedure involves the insertion of a small gauge needle (27-gauge [G] to 33G) and the injection of 0.05 mL of fluid.

Needle insertion and drug injection can elevate the intraocular pressure (IOP) in both the short term and the long term; this constitutes the main complication of IVI. IOP peaks and IOP fluctuations can be especially harmful to patients who already have glaucoma or retinal diseases [1–3]. For this reason, several studies have measured IOP immediately after IVI (post-IOP) to confirm the short-term IOP changes caused by needles of different sizes [4, 5]. Because penetration force and IOP elevation are related to pain, penetration force and pain scores have also been measured for different needle sizes [6–9].

Real-time IOP measurement can increase the understanding of how each step of the surgical procedure affects IOP [10, 11]. For example, real-time IOP measurement during laser in situ keratomileusis (LASIK) has been performed to determine which surgical step increased the IOP [12–15]. In the case of IVI, however, previous studies did not monitor the IOP change and only measured post-IOP or needle insertion force. Christensen et al. measured IOP in real-time using five different types of needles, but IOP change during scleral perforation was not investigated [16]. Moreover, they had a small sample size (30 porcine eyes).

In other previous studies, IVI was performed manually, and the effect of needle insertion speed was not discussed (Table 1). According to van Gerwen at al. and Jiang et al., the needle-tissue interaction force is affected by the needle insertion speed, so the magnitude of the insertion force and the corresponding IOP changes can differ from clinician to clinician [17, 18]. Therefore, an experiment with constant insertion speeds and injection rates is required for a systematic investigation.

**Table 1:** Summary of previous studies on insertion force and intraocular pressure (IOP) changes during intravitreal injection.

Ref.	Subject	# of subject	Needle size [G]	Insertion force	Insertion speed [mm/s]	Injection rate [mL/s]	IOP
Pang et al. [5]	In-vivo human	65	30,32	-	-	-	Post IOP
Hubschman et al. [7]	In-vivo human	205	26,27,29,30	Pain score	-	-	-
Aderman et al. [6]	In-vivo human	98	30,33	Pain score	-	-	-
Kotliar et al. [4]	In-vivo human	22	27	-	-	-	Post IOP
Pulido et al. [8]	Ex-vivo porcine	12	27,30,31	Peak force	0.25	-	-
Christensen et al. [16]	Ex-vivo porcine	30	19,25,27,30,32	Real-time	-	-	Real-time
The present study	Ex-vivo porcine	90	27,30,33	Real-time	1, 2, 5	0.01, 0.02, 0.05	Real-time

The systematic investigation can be utilized to develop novel devices and simulation for IVI. For example, Ullrich et al. proposed a robotic injection device combined with a image processing algorithm for pupil tracking, but the guidelines on insertion speed or injection rate were not discussed [19]. Moreover, the systematic data can be utilized to validate needle insertion and drug injection simulation, which requires comparison between actual results and simulated results [20, 21].

In this study, an experimental device that automates needle insertion and drug injection was designed to reproduce IVI procedures. While IVI is performed with constant insertion speeds and injection rates, the needle insertion force and the IOP of the eye are recorded in real-time. The aim of the study was to investigate the effects of needle size, insertion speed, and injection rate on IOP by an ex vivo experiment on 90 porcine eyes.

## Materials and methods

### Design of an automatic IVI device

**Fig 1:**
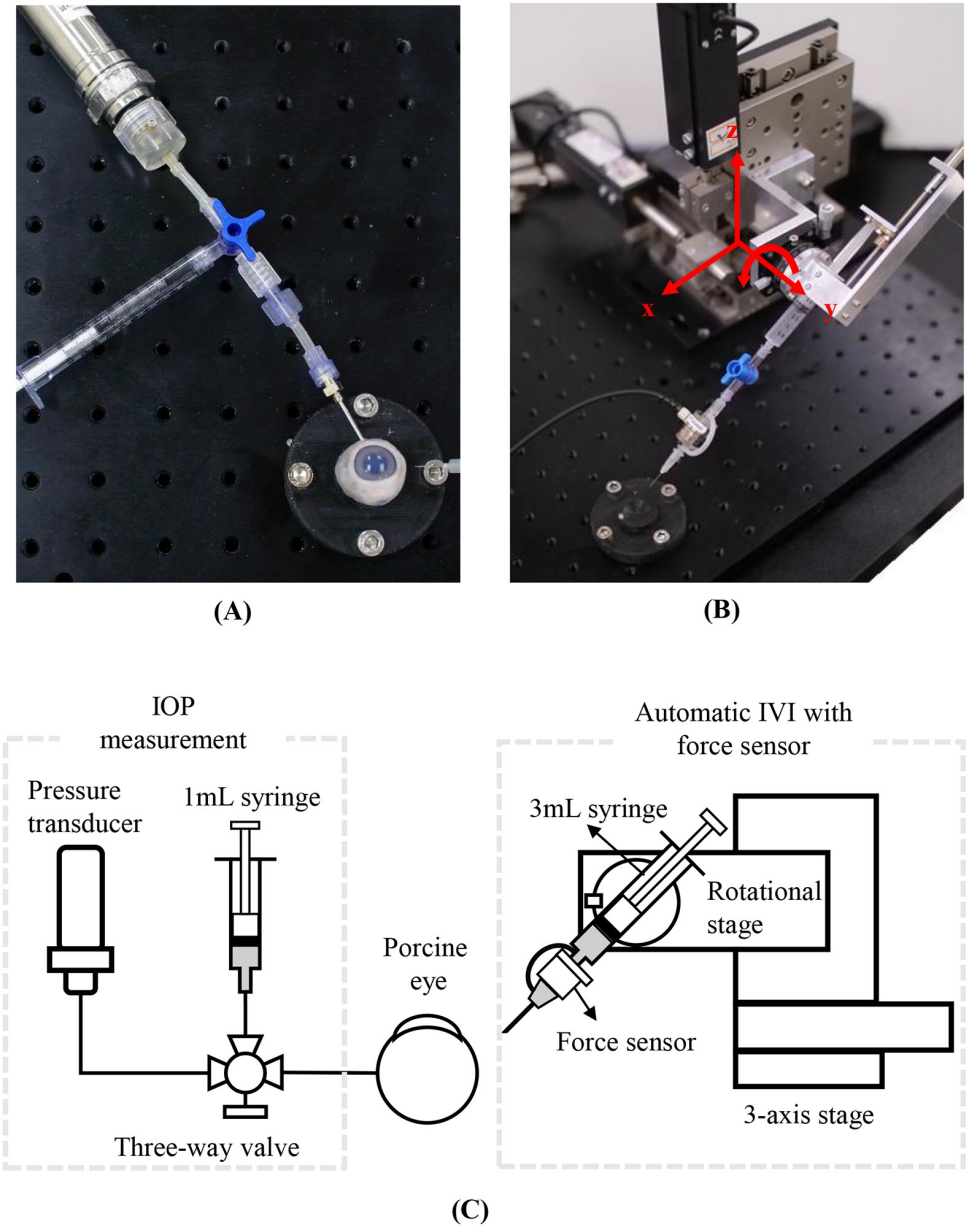
The automatic IVI device and experimental setup. A: A porcine eye, 17-gauge needle for IOP measurement, syringe for maintaining initial IOP, and a pressure transducer, B: an automatic injection device with a motor and ball screw, and 3-axis stage for insertion, and C: a schematic explanation of the system.

The automatic IVI device used in this study included a 3-axis motorized stage with a travel range of 50 mm, a maximum velocity of 20 mm/s, and a minimum step size of 0.05 μm (LNR50K1/M, Thorlabs, New Jersey, United States), a rotational stage (PR01/M, Thorlabs, New Jersey, United States), a 6-axis force sensor with a force range of 12 N and a resolution of 0.003 N (Nano 17 SI-12-0.12, ATI Industrial Automation, Apex, NC, USA), and a DC motor (DCX08M, Maxon, Sachseln, Switzerland) with a gear ratio of 256:1 (Fig 1B).

Needles were attached to the force sensor and then connected to a 3 mL syringe through a silicon tube. The plunger of the syringe was connected to the ball screw with a lead of 1 mm so that the fluid could be injected by the rotational motion of the motor. Considering the size of the syringe, the lead of the ball screw, and the maximum speed of the motor, the maximum rate of injection was 0.08 mL/s. For measuring IOP, a 17G needle was inserted into the eye and was directly connected to a pressure transducer (PXM409-170HGUSBH, Omega Engineering, Connecticut, United States) (Fig 1A).

### Specimen and experiment protocol

Ninety porcine eyes were obtained from a slaughterhouse (Yeon-il Livestock, Pohang, Korea). The experiment was performed within 7 h after enucleation. All experiments were performed in accordance with the guidelines of the Animal Care and Use Committee (IACUC) at POSTECH. The approval procedure was exempt because the porcine eyes were obtained as a byproduct of the slaughter process and not harvested for this experiment.

Because enucleated eyes could not maintain the normal IOP of 15 mmHg, a 17G needle was inserted, and saline solution was manually injected through the needle to ensure the normal IOP. The connection between the eyeball and the syringe was blocked by manipulating the 3-way valve in Fig 1C. The insertion of the 17G needle was maintained during the experiment to measure IOP in real-time.

The steps followed during the overall experimental process were: First, the rotational stage was manually adjusted so that the experimental needle was perpendicularly inserted into the eye. Second, the experimental needle was connected to the force sensor, and the air inside the needle was ejected. Third, the needle was inserted 10 mm into the eye, and 0.05 mL of saline solution was injected with a constant insertion speed and injection rate. Finally, the needle was extracted. During the experiment, the insertion force and the IOP were recorded at a sampling rate of 200 Hz in real-time.

Three different sizes of 1/2-inch needles (27, 30, and 33G), three different insertion speeds (5, 2, and 1 mm/s), and three different injection rates (0.05, 0.02, and 0.01 mL/s) were used in the experiment. Each trial employed one porcine eye and one needle, and both, eye and needle, were replaced for the next trial. All recorded data were analyzed using MATLAB software (R2019b, MathWorks), including data used for data fitting and hypothesis testing. The two-sample t-test was used to compare the subgroups, and a p-value less than 0.05 was considered to be significant.

## Results

**Fig 2:**
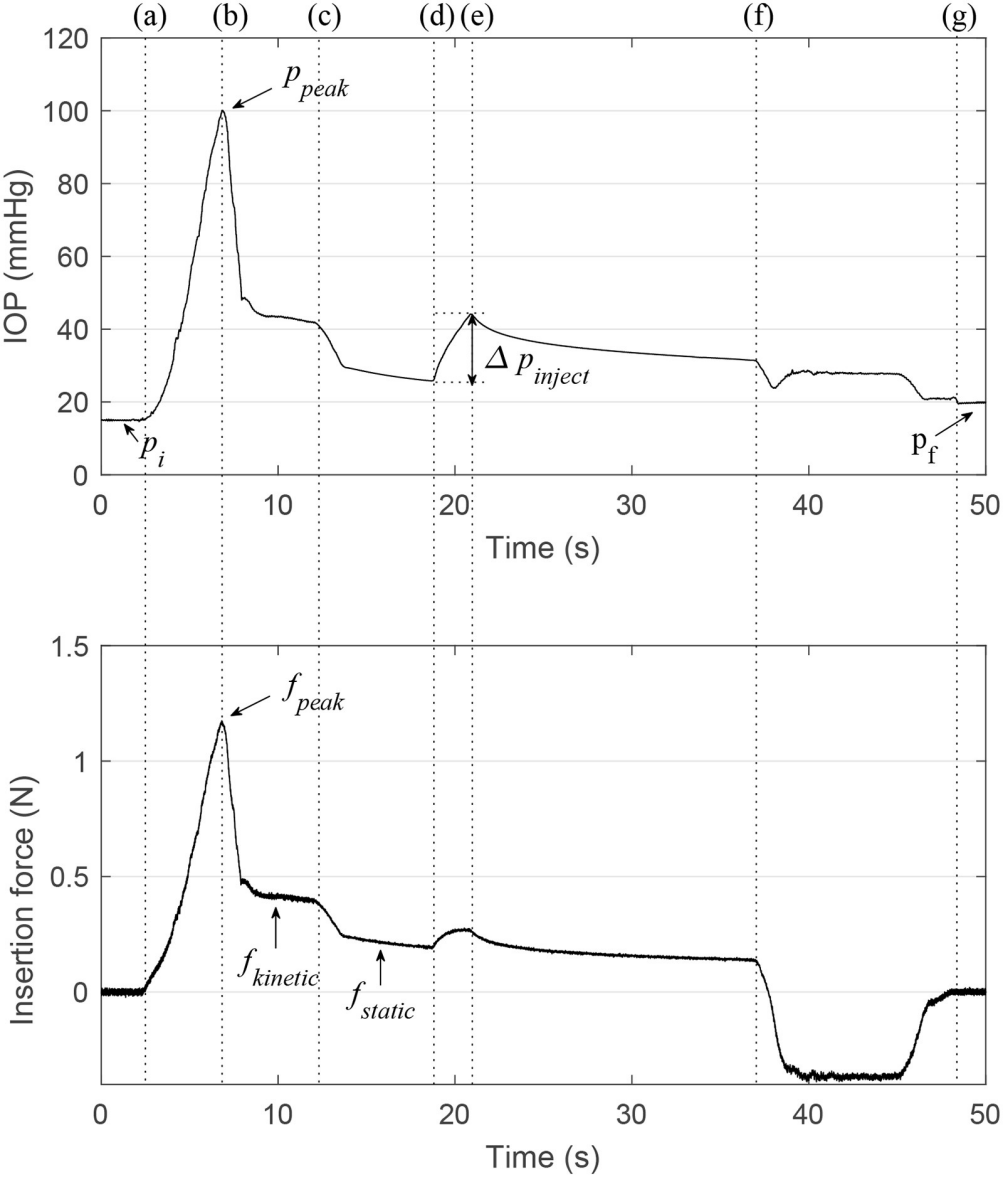
Typical intraocular pressure and insertion force profiles during intravitreal injection. The needle contacts the eye (a), penetrates the sclera (b), and stops (c). The injection is initiated (d) and ended (e). The needle extraction is initiated (f) and ended (g).

Fig 2 shows typical IOP and force profiles during IVI. As the insertion force increased, the IOP also increased until the needle penetrated the sclera (Fig 2a and 2b). After penetration, the insertion induced kinetic friction or static friction, depending on the velocity of the needle (Fig 2b–2d). The injection of saline solution caused an increase in IOP and, after the injection, the IOP decreased over time (Fig 2d–2f). We denoted the initial IOP as *p*_*i*_, peak IOP and force for sclera puncture as *p*_*peak*_ and *f*_*peak*_, respectively, IOP elevation during injection as Δ*p*_*inject*_, and final IOP as *p*_*f*_.

The different experimental groups were noted as G-x-y, where “x” represented the needle gauge size, and “y” represented the insertion speed. For example, G-27-5 represents a 27G needle with an insertion speed of 5 mm/s. Experimental values are represented as mean ± SD.

### IOP elevation and peak force

**Fig 3:**
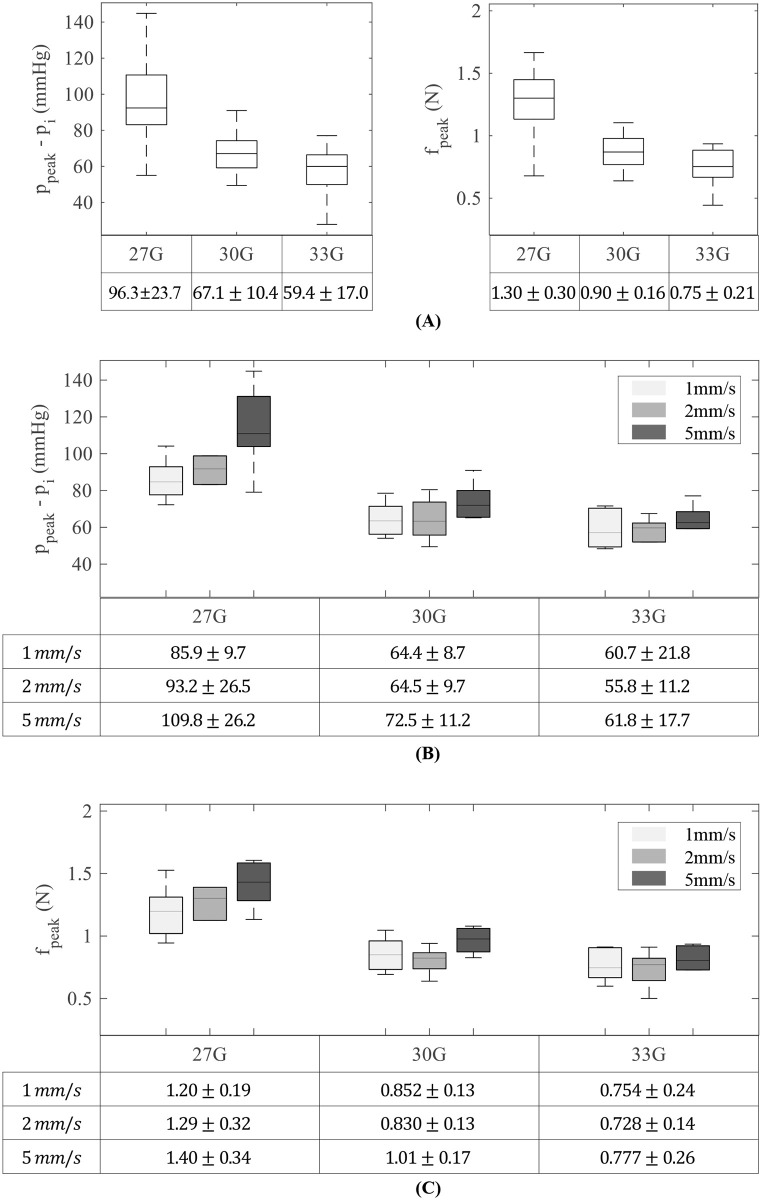
Pressure elevation and peak force in the insertion stage. According to A: needle gauge and B, C: insertion speed. Each subgroup contains 30 samples in A, and 10 samples in B and C.

The mean of the initial IOP (*p*_*i*_) was 15 ± 1.2 mmHg, with a maximum value of 18.4 mmHg and a minimum value of 13.1 mmHg. Fig 3 shows the peak force and pressure change when the needle penetrates the sclera.

The size of the needle affected both pressure elevation and insertion force (Fig 3A). The mean IOP elevation was 96.3 ± 23.7, 67.1 ± 10.4, and 59.4 ± 17.0 mmHg in 27G, 30G, and 33G, respectively. IOP elevation due to the 33G needle was smaller than that due to other sizes (p < 0.001 with 27G and p = 0.039 with 30G). The mean *f*_*peak*_ was 1.30 ± 0.30, 0.90 ± 0.30, and 0.75 ± 0.21 N for 27G, 30G, and 33G needles, respectively. *f*_*peak*_ for 33G needles was also smaller than that for the other sizes (p < 0.001 with 27G and p = 0.004 with 30G needles).

The effect of insertion speed was investigated within each group (Fig 3B and 3C). The mean pressure elevation was 85.9 ± 9.7, 93.2 ± 26.5, and 109.8 ± 26.2 mmHg in G-27-1 to G-27-5, respectively. The p-values for pressure elevation were 0.4256 between G-27-1 and G-27-2, 0.1776 between G-27-2 and G-27-5, and 0.0149 between G-27-1 and G-27-5. The effect of insertion speed diminished as the needle size decreased: 64.4 and 72.54 mmHg in G-30-1 and G-30-5, respectively (p = 0.0865), and 60.7 and 61.8 mmHg in G-33-1 and G-33-5, respectively (p = 0.8979).

**Fig 4:**
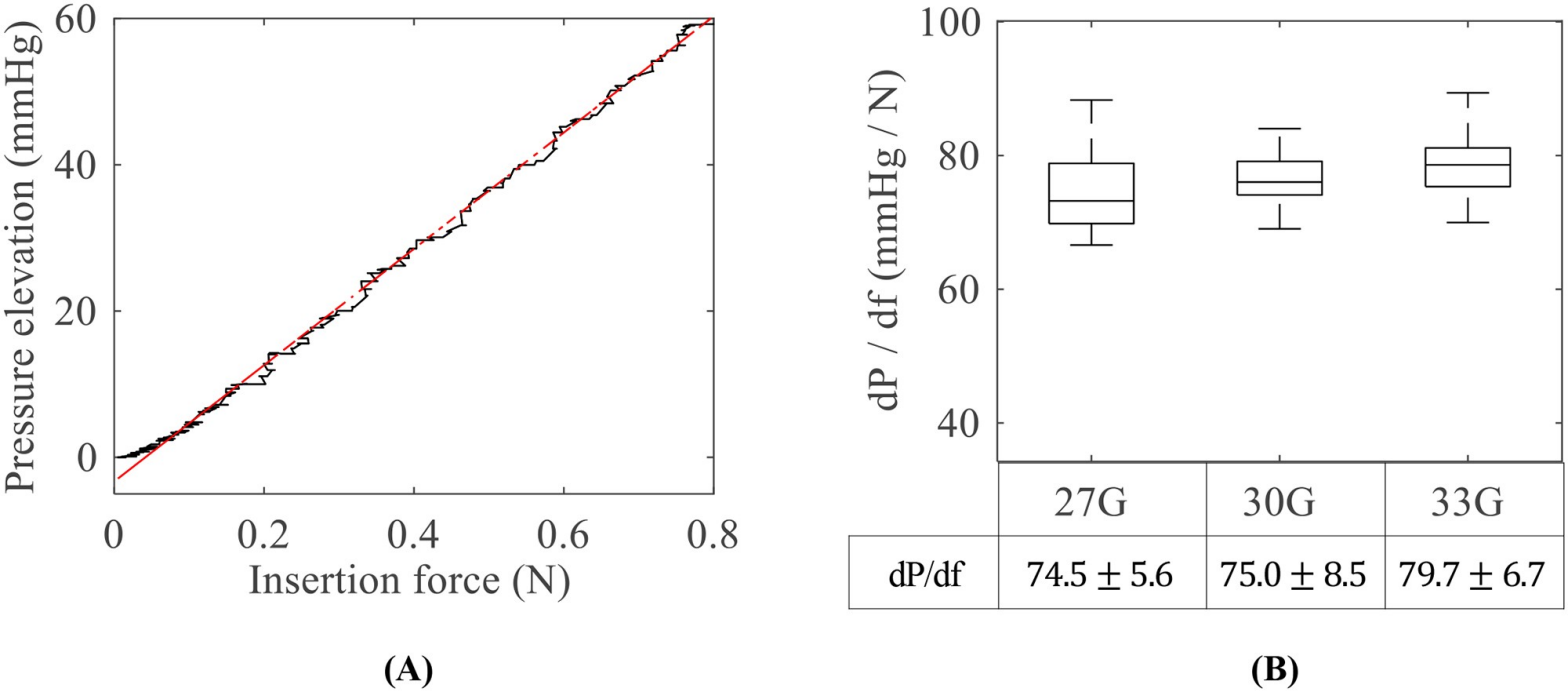
Relationship between pressure and force. A: Linear relationship between pressure elevation and insertion force, and B: slope values according to needle size. Each subgroup contains 30 samples.

The IOP linearly increased as the needle force increased (Fig 4A). The two parameters were fitted to a linear function; the function’s slope value indicates the IOP elevation per unit of force. Fig 4B shows the slope values according to the needle size. The mean slope value was 74 ± 5.6 mmHg/N in 27G, 75.0 ± 8.5 mmHg/N in 30G, and 79.7 ± 6.7 mmHg/N in 33G. The slope value increased as the needle size decreased, and the p-values were 0.698 (between 27G and 30G), 0.0224 (between 30G and 33G), and 0.0014 (between 27G and 33G). However, because the peak force was significantly small in 33G, 33G needle showed the smallest increase in IOP compared to the other needles.

### Friction force

After sclera puncture, the friction force between the sclera and the needle was observed (Fig 2b–2d). Friction force can be divided into kinetic friction (Fig 2b and 2c) and static friction (Fig 2c and 2d). The median value of the force in each range was defined as *f*_*kinetic*_ and *f*_*static*_.

**Fig 5:**
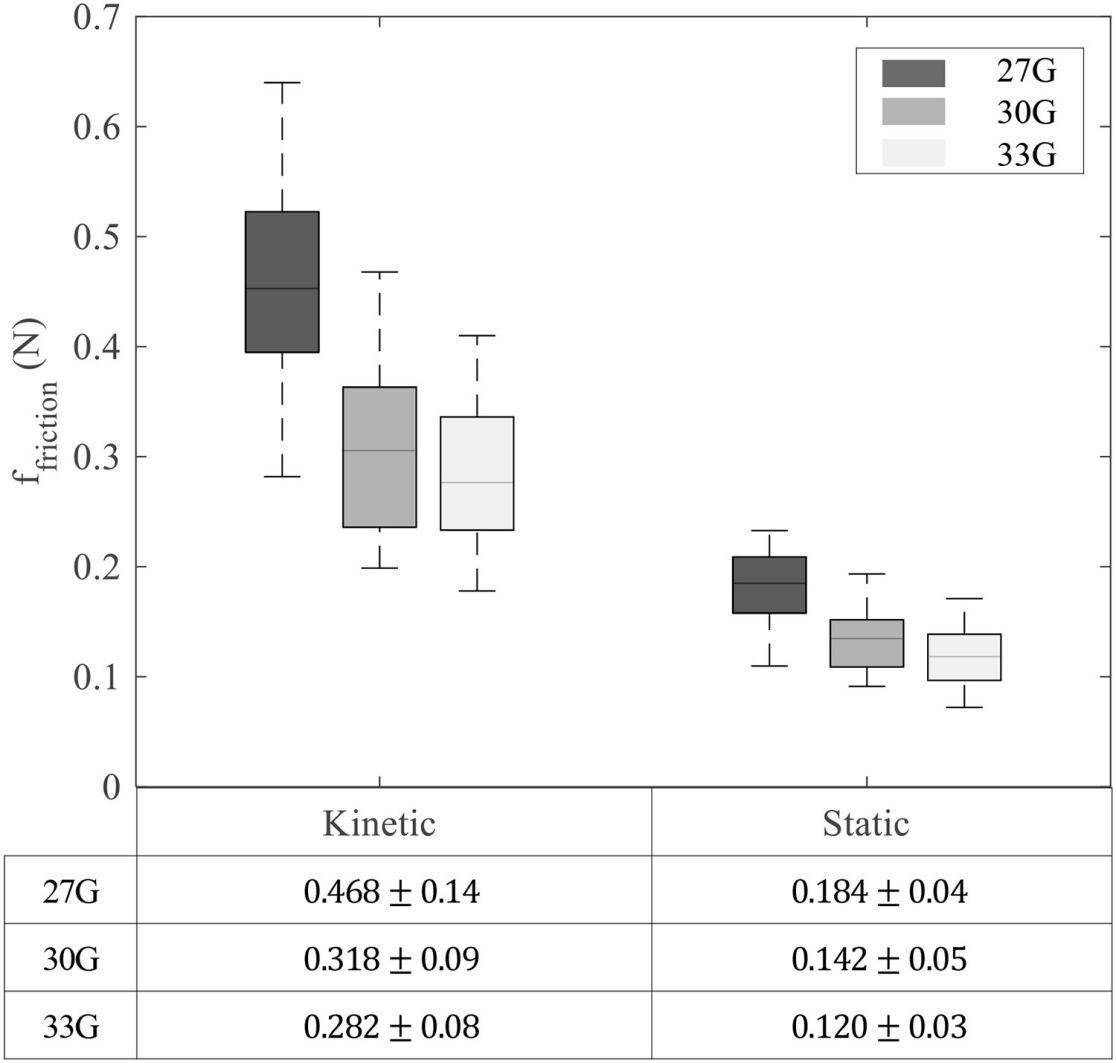
Kinetic friction according to needle size and static friction when the needle stopped. Each subgroup contains 30 samples.

Both *f*_*kinetic*_ and *f*_*static*_ decreased as the needle size decreased (Fig 5). The mean *f*_*kinetic*_ was 0.468 ± 0.14, 0.318 ± 0.09, and 0.282 ± 0.08 N for 27G, 30G, and 33G, respectively. The mean *f*_*static*_ was 0.184 ± 0.04, 0.142 ± 0.05, and 0.120 ± 0.03 N for 27G, 30G, and 33G, respectively. The friction force was significantly higher when using the 27G needle than when using smaller needles (p < 0.001).

### Injection rate and relaxation

**Fig 6:**
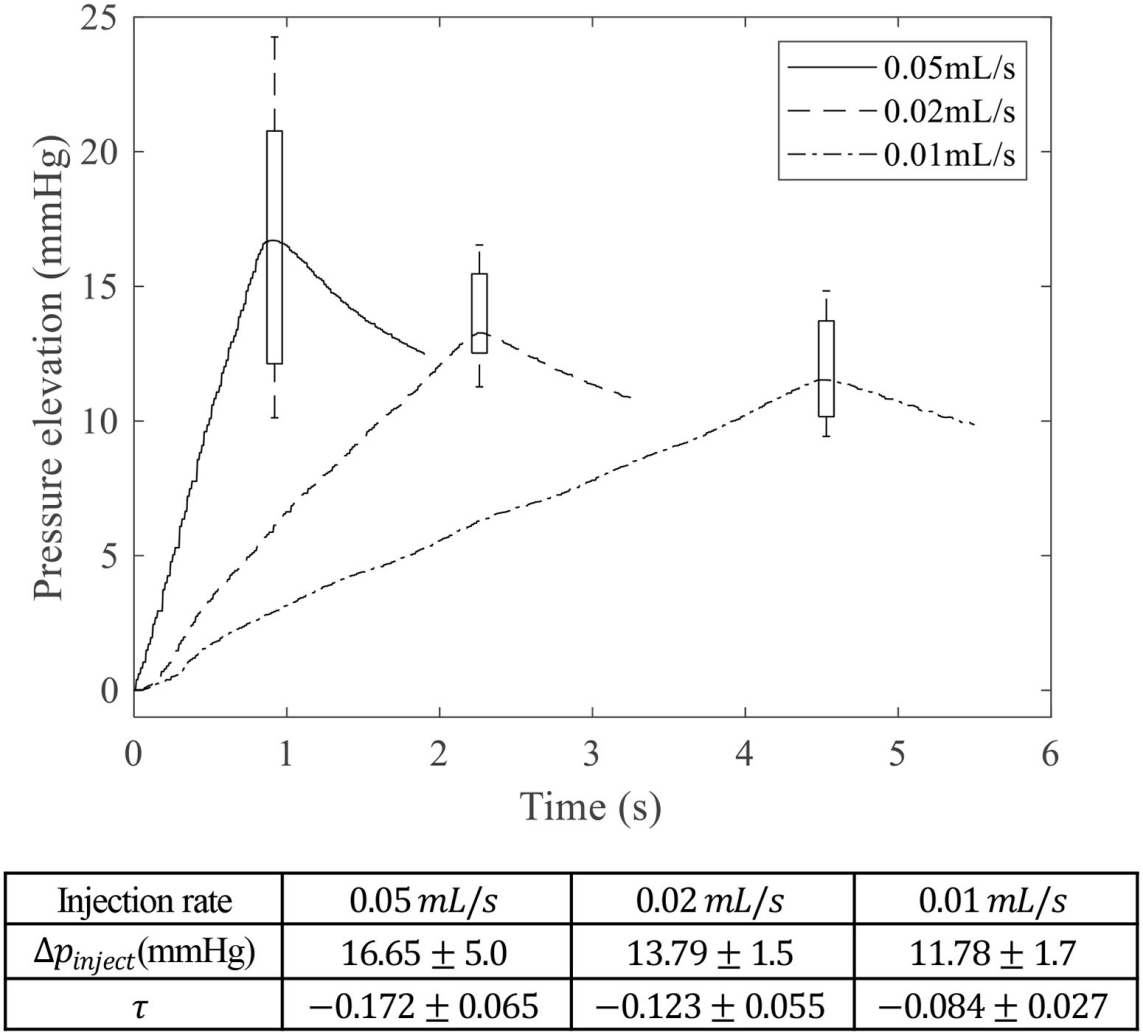
Pressure elevation and relaxation according to injection rate. The maximum pressure values were also represented by a Whisker plot. Each subgroup contains 30 samples.

The injection volume was the same (0.05 mL), but the pressure elevation was different depending on the injection rate (Fig 6). The pressure elevation was 16.65 ± 5.0, 13.79 ± 1.5, and 11.78 ± 1.7 mmHg in 0.05, 0.02, and 0.01 mL/s, respectively. The p-value was 0.0265 between 0.05 and 0.02 mL/s, and p-values were smaller than 0.001 in the other group comparisons. The pressure elevation during injection was significantly higher in the faster injection group than in the slower injection group.

After the injection, a pressure relaxation occurred. The pressure profile was fitted to the first exponential function (*p* = a*exp(τ*t)). *τ* was defined as the relaxation coefficient. The relaxation coefficients were -0.172 ± 0.065 for 0.05 mL/s, -0.123 ± 0.055 for 0.02 mL/s, and 0.084 ± 0.027 for 0.01 mL/s. This means that the level of relaxation increased as the injection speed increased.

### IOP change after finishing IVI

**Fig 7:**
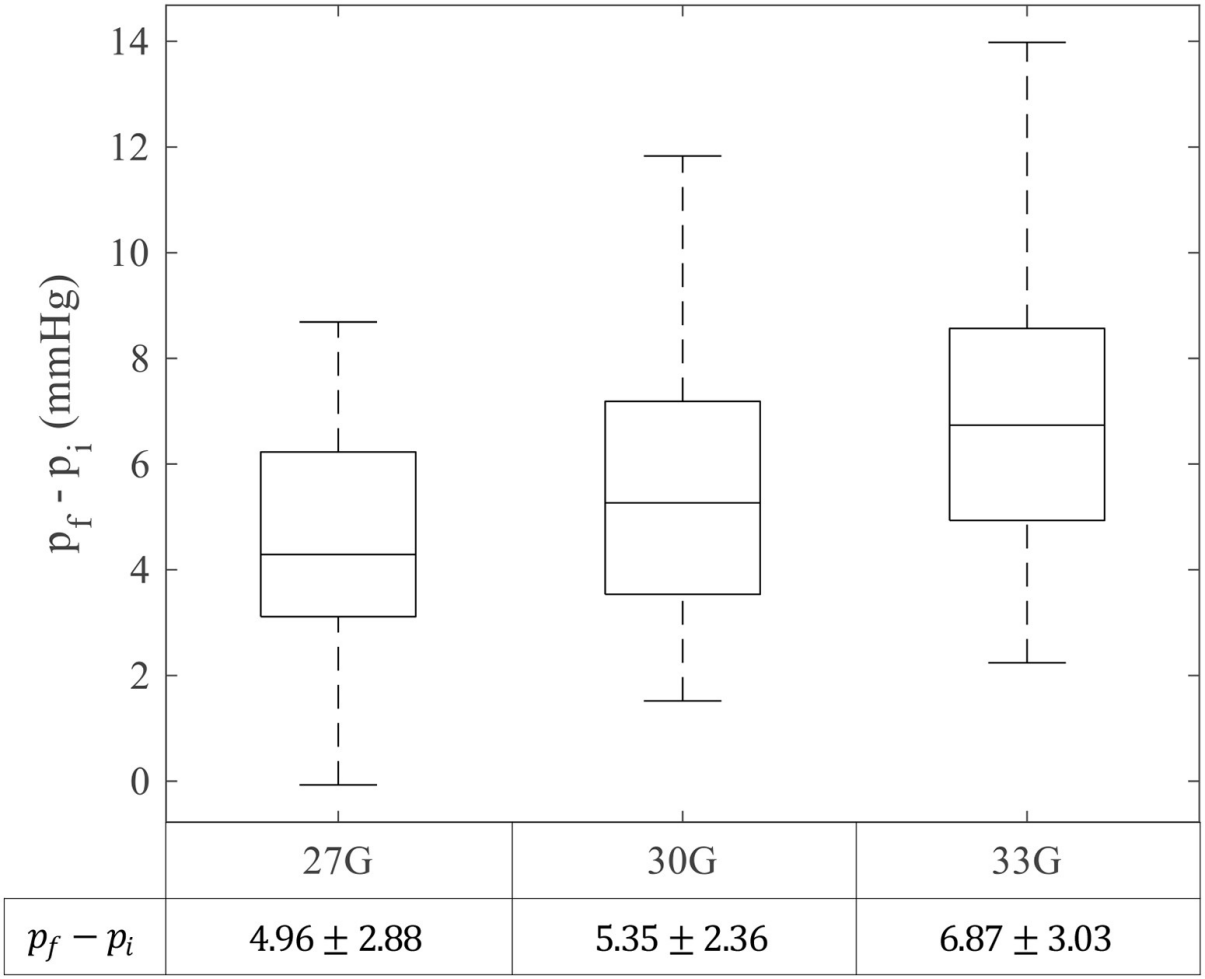
Pressure elevation after ending intravitreal injection. Each subgroup contains 30 samples.

The IOP difference between the initial IOP (*p*_*i*_) and the final IOP (*p*_*f*_) was calculated according to the needle size (Fig 7). The IOP elevations were 4.96 ± 2.88 mmHg for 27G, 5.35 ± 2.36 mmHg for 30G, and 6.87 ± 3.03 mmHg for 33G. The IOP elevation increased as the needle size decreased, and the p-values were 0.573 (between 27G and 30G), 0.0336 (between 30G and 33G), and 0.0151 (between 27G and 33G). It should be noted that, in our experiment, a cotton swab was not placed over the injection site, although it is a common procedure to prevent reflux.

## Discussion

Needle insertion force and reflux decreased as the needle size decreased, which is consistent with the results of previous research. Christensen et al. and Pulido et al. showed that the 27G needle requires a larger penetration force than the 30G needle [8, 16]. Our experimental results also showed that a smaller needle could reduce not only the penetration force and the peak IOP, but also the friction force. Moreover, because the difference between initial IOP and final IOP increased as the needle size decreased, the use of a 33G needle can reduce vitreous reflux, which is consistent with the results of a previous study [5].

Our study found that faster needle insertion resulted in a larger penetration force. This phenomenon was more significant in the 27G needle, and the effect of speed was reduced as the needle size decreased. Therefore, insertion speed must be carefully controlled when using a 27G needle. During the insertion of the 33G needle at a speed of 1 mm/s, needle bending frequently occurred, which resulted in a change in insertion angle or even in insertion failure. The cases of insertion failure were not included in the analysis, but the cases of needle bending were included.

Willekens et al. showed that the injection speed does not affect drug dispersion [22]. On the other hand, in terms of pressure change, injection speed is related to the pressure elevation. The average IOP elevation was 16.65 mmHg in 0.05 mL/s, and 11.78 mmHg in 0.01 mL/s, and the p-value was lesser than 0.001 between the two groups. This phenomenon could be explained by the viscoelastic properties of the human eye. Slow injection can give the eye time for relaxation [23, 24]. Therefore, slow injection of the drug is recommended to avoid an unwanted IOP peak.

The first limitation of the presented experiment is the use of porcine eyes. The compressive modulus of the porcine sclera is three times higher than that of the human sclera, although the thickness is similar between both tissues [25, 26]. The penetration force required could be larger in porcine eyes than in human eyes [8]. The second limitation is the boundaries constraining the studied eyeballs. The posterior surface of the eyeball is covered with extraocular muscles, but the enucleated eyeballs were freely bulged without boundary constraints. An in vivo experiment is required in further studies to validate the results presented here.

## Conclusion

In this study, insertion force and IOP change during IVI were measured in real-time using 90 porcine eyeballs. The results show that IOP elevation can vary depending on the needle size, the insertion speed, and the injection rate. We believe that the systematic investigation of needle insertion force and IOP elevation during IVI can be used to develop novel devices for drug injection, and surgical simulators for IVI.
